# Genome-wide comparative analysis of NBS-encoding genes between *Brassica* species and *Arabidopsis thaliana*

**DOI:** 10.1186/1471-2164-15-3

**Published:** 2014-01-03

**Authors:** Jingyin Yu, Sadia Tehrim, Fengqi Zhang, Chaobo Tong, Junyan Huang, Xiaohui Cheng, Caihua Dong, Yanqiu Zhou, Rui Qin, Wei Hua, Shengyi Liu

**Affiliations:** Key Laboratory of Biology and Genetic Improvement of Oil crops, the Ministry of Agriculture, Oil Crops Research Institute of the Chinese Academy of Agricultural Sciences, Wuhan, 430062 China; Engineering Research Center of Protection and Utilization for Biological Resources in Minority Regions, South-Central University for Nationalities, Wuhan, 473061 China

**Keywords:** Brassica species, Disease resistance gene, Nucleotide binding site, Tandem duplication, Whole genome duplication

## Abstract

**Background:**

Plant disease resistance (R) genes with the nucleotide binding site (NBS) play an important role in offering resistance to pathogens. The availability of complete genome sequences of *Brassica oleracea* and *Brassica rapa* provides an important opportunity for researchers to identify and characterize NBS-encoding R genes in *Brassica* species and to compare with analogues in *Arabidopsis thaliana* based on a comparative genomics approach. However, little is known about the evolutionary fate of NBS-encoding genes in the *Brassica* lineage after split from *A. thaliana*.

**Results:**

Here we present genome-wide analysis of NBS-encoding genes in *B. oleracea*, *B. rapa* and *A. thaliana*. Through the employment of HMM search and manual curation, we identified 157, 206 and 167 NBS-encoding genes in *B. oleracea*, *B. rapa* and *A. thaliana* genomes, respectively*.* Phylogenetic analysis among 3 species classified NBS-encoding genes into 6 subgroups. Tandem duplication and whole genome triplication (WGT) analyses revealed that after WGT of the *Brassica* ancestor, NBS-encoding homologous gene pairs on triplicated regions in *Brassica* ancestor were deleted or lost quickly, but NBS-encoding genes in *Brassica* species experienced species-specific gene amplification by tandem duplication after divergence of *B. rapa* and *B. oleracea*. Expression profiling of NBS-encoding orthologous gene pairs indicated the differential expression pattern of retained orthologous gene copies in *B. oleracea* and *B. rapa*. Furthermore, evolutionary analysis of CNL type NBS-encoding orthologous gene pairs among 3 species suggested that orthologous genes in *B. rapa* species have undergone stronger negative selection than those in *B .oleracea* species. But for TNL type, there are no significant differences in the orthologous gene pairs between the two species.

**Conclusion:**

This study is first identification and characterization of NBS-encoding genes in *B. rapa* and *B. oleracea* based on whole genome sequences. Through tandem duplication and whole genome triplication analysis in *B. oleracea*, *B. rapa* and *A. thaliana* genomes, our study provides insight into the evolutionary history of NBS-encoding genes after divergence of *A. thaliana* and the *Brassica* lineage. These results together with expression pattern analysis of NBS-encoding orthologous genes provide useful resource for functional characterization of these genes and genetic improvement of relevant crops.

**Electronic supplementary material:**

The online version of this article (doi:10.1186/1471-2164-15-3) contains supplementary material, which is available to authorized users.

## Background

Plants are surrounded by a large number of invaders including bacteria, fungi, nematodes and viruses, and some of them have successfully invaded crop plants and cause diseases which result in deterioration of crop quality and yield. In order to cope with disease attacks, the plants have developed multiple layers of defense mechanisms. Plant disease resistance (R) genes which specifically interact/recognize with corresponding pathogen avirulence (*avr*) genes are considered as plant genetic factors of a major layer. The interactions of this gene-for-gene (or genes-for-genes) manner activate the signal transduction cascades that turn on complex defense responses against pathogen attack and this is called incompatible interaction [1]. The interaction between a host species and a pathogenic species is dynamic where a host variety often lost the R gene-dependent resistance due to its pathogen race evolution for a virulent gene and thus a new R gene was selected against this new race [2]. R genes provide innate immunity whereas outcomes of defense responses lacking R genes are partial resistance [3]. Therefore, identification of R genes is crucial for resistant variety development and relevant mechanism investigation.

To date, more than one hundred R genes, which was reported in PRGdb (http://prgdb.crg.eu/wiki), were functionally identified and comprise a super family in plants [4]. Sequence composition analysis of R genes indicate that they share high similarity and contain seven different conserved domains like NBS (nucleotide-binding site), LRR (leucine rich repeat), TIR (Toll/Interleukin-1 receptor), CC (coiled-coil), LZ (leucine zipper), TM (transmembrane) and STK (serine-threonine kinase). Based on domain organization, R gene products can be categorized into five major types: TNL (TIR-NBS-LRR), CNL (CC-NBS-LRR), RLK (Receptor like kinases), RLP (Receptor like proteins) and Pto (a Ser/Thr kinase protein) [1, 5, 6]. Most of the R genes in plant kingdom are members of NBS-LRR (nucleotide-binding site-leucine rich repeat) proteins. ‘NBS’ and ‘LRR’ domains play different roles in plant-microbe interaction, where the former have the ability to bind and hydrolyze ATP or GTP and the latter is involved in protein–protein interactions [7]. NBS-LRR proteins in plants share sequence similarity with the mammalian NOD-LRR containing proteins which play a role in inflammatory and immune responses. On the basis of presence or absence of N-terminal domains (TOLL/ interleukin-1 receptor (TIR) and the coiled-coil (CC) motif), NBS-LRR class can be further divided into two major types, TNL (TIR-NBS-LRR) and CNL (CC-NBS-LRR). TNL type share homology with the Drosophila toll and human interleukin-1 receptor (TIR). The two types show divergence in their sequence and signaling pathways. Several partial NBS-LRR variants like TIR, TIR-NBS (TN), CC, CC-NBS(CN) and NBS (N) have also been identified in plant species [6, 8, 9].

Recent whole genome sequence data enabled the genome wide identification, mapping and characterization of candidate NBS-containing R genes in economically important plants. For example, the approximate arrays of 159 NBS-encoding R genes in *A. thaliana*[10], 581 in *Oryza sativa*[11], 400 in *Populus trichocarpa*[12], 333 in *Medicago truncatula*[13], 54 in *Carica papaya*[14], 534 in *Vitis vinifera*[15] and 158 in *Lotus japonicas*[16] have been identified. Earlier genome-wide studies have demonstrated that TNL subfamily is abundant in dicots while absent in cereals (monocots) [17]. The presence of the full length of TNL and CNL types in the common ancestor (mosses) of both angiosperms and gymnosperms and exceptional presence of truncated domains of TN or TX type proteins in cereals indicate that the TNL class might have been lost in monocot plants [9, 18]. On the chromosomes, the NBS-LRR R genes are arranged in clusters. The genes in the clusters could be homogenous (often tandem duplicated from single ancestor gene) or heterogenous (with different protein domains) [19–21]. However, the variation of the number and sequences of the R genes presented in the *Brassica* lineage since split from the *Arabidopsis* lineage and their distributions in chromosomes are unknown.

The genera *Brassica* and *Arabidopsis*, both belong to the mustard family *Brassicaceae* (Cruciferae), are a model plant and a model crop, respectively. The two genera shared a latest and obviously detectable alpha genome duplication event before their divergence ~20 million years ago (MYA) and subsequently *Brassica* ancestor underwent a whole genome triplication event (common to the tribe *Brassicaceae*) ~16 MYA [22–25]. In *Brassica*, interspecific cytogenetic relationship between important crops (oilseed and vegetables) is well-described by a “U” triangle where each two diploid species [*B.rapa* (AA, 2n = 20), *B. oleracea* (CC, 2n = 18) and *B. nigra* (BB, 2n = 16)] formed a tetraploidy species [*B.napus* (AACC, 2n = 38), *B. juncea* (AABB, 2n = 36) or *B. carinata* (BBCC, 2n = 34)] [26]. This well-established phylogenetic relationship provides a chance to trace evolution of the R genes between wild plants and their relative crops. The present study is to identify R genes on genome-wide scale in *B. oleracea* and *B. rapa* and provide insights into their evolutionary history and disease resistance.

## Methods

### Data resource

*Arabidopsis thaliana*, *Brassica rapa* and *Brassica oleracea* genomic and annotation data was downloaded from the TAIR10 (http://www.arabidopsis.org) [27], the BRAD database (http://brassicadb.org/brad/) [28] and the Bolbase database (http://ocri-genomics.org/bolbase) [29], respectively. *Theobroma cacao* genomic data was downloaded from http://cocoagendb.cirad.fr/, *Populus trichocarpa* genomic data was downloaded from JGI database (http://www.phytozome.net/poplar.php)*, Vitis vinifera* genomic data was downloaded from http://www.genoscope.cns.fr/externe/GenomeBrowser/Vitis/, *Medicago truncatula* genomic data was downloaded from http://www.medicago.org/. The Hidden Markov Model (HMM) profiles of NBS and TIR domain (PF00931 and PF01582) were retrieved from Pfam 26.0 (http://Pfam.sanger.ac.uk) [30]. *B. rapa* and *B. oleracea* illumina RNA-seq data were obtained from the Gene Expression Omnibus (GEO) database with accession numbers GSE43245 and GSE42891 respectively.

### Identification of *B. oleracea*genes that encode NBS domain and NBS-associated conserved domains

In the draft genome of *B. oleracea*, NBS-encoding genes were identified through Hidden Markov Model (HMM) profile corresponding to the Pfam NBS (NB-ARC) family PF00931 domain using HMMER V3.0 programme with “trusted cutoff” as threshold [31]. From the selected protein sequences screened through NBS domain, high quality sequences were aligned through CLUSTALW [32] and used to construct *B. oleracea* specific NBS profile using the “hmmbuild” module by HMMER V3.0 programme. With this model final set of NBS-encoding proteins were identified and only 157 proteins were selected as NBS candidate genes with stringent parameters. The NBS R-gene family is subdivided into different groups based on the structure of the N-terminal and C-terminal domains of the protein. For the identification of N-terminal and C-terminal domains of NBS-encoding genes, we used HMMPfam and HMMSmart for detection. We further employed PAIRCOIL2 [33] (P score cut-off of 0.025) and MARCOIL [34] programs with a threshold probability of 90 to confirm Coiled-Coil (CC) motif. From the result generated by these programs, we selected overlapping sequences as candidate genes with CC motif. We used same procedures to identify genes that contain TIR domain only and excluded the NBS-encoding genes as TIR-X genes. NBS-encoding genes in *A. thaliana* and *B. rapa* have been reported earlier but in order to get the latest NBS-encoding genes in these two species for our comparative analysis, we followed the same procedures to screen NBS candidate genes in *B. rapa* and *A. thaliana* for consistency.

### Assigning the location of NBS-encoding genes to *B. oleracea*and *B. rapa*genome

The physical position of NBS-encoding genes was mapped to the 9 and 10 pseudo-molecular chromosomes of *B. oleracea* and *B. rapa* using GFF file which was downloaded from Bolbase [29] and BRAD [28] database respectively. After that, we used in-house perl script to draw graphic potryl of NBS-encoding genes on pseudo-molecular chromosomes with SVG module [35].

### Identification of tandem duplicated arrays

To detect the generated mechanism of NBS-encoding genes, BLASTP program [36] was employed to identify the tandem duplicated genes using protein sequences with E-value cutoff ≤ 1e-20, and one unrelated gene was allowed within a tandem array.

### Alignment and phylogenetic analysis of NBS-encoding genes

According to location of conserved domains for NBS (Nucleotide-binding Site) in complete predicted NBS protein sequences, conserved domain sequences of NBS-encoding genes were extracted and aligned using the programme Clustal W [32] with default options for the phylogenetic analysis among 3 species. The poor alignment sequences were excluded by manually curation using Jalview [37]. The resulting sequences were used to construct a phylogenetic tree using Maximum Likelihood (ML) method in MEGA 5.0 [38] with 1000 replications.

### Orthologous gene pairs between *B. rapa*, *A. thaliana*and *B. oleracea*

Orthologous gene pairs provide information about the evolutionary relationship between different species. In our study, we used two steps to detect gene pairs precisely. First, MCscan programme [39] was employed to identify orthologous regions with the parameters (e = 1e-20, u = 1 and s = 5. Parameter of s = 5) between *B. rapa*, *A. thaliana and B. oleracea* genomes. Second, after extracting orthologous regions that contained NBS-encoding genes, orthologous gene pairs of NBS-encoding genes were extracted.

### Non-synonymous/synonymous substitution (Ka/Ks) ratios of gene pairs between *B. rapa*, *A. thaliana*and *B. oleracea*

For the estimation of selection mode for the NBS-encoding genes among *B. oleracea*, *B. rapa* and *A. thaliana*, the ratio of the rates of nonsynonymous to synonymous substitutions (Ka/Ks) of all orthologous gene pairs were calculated for each branch of the phylogenetic tree using PAML software [40]. For each subtree of NBS orthologous gene pairs among 3 species , model 1 with a free Ka/Ks ratio was calculated separately for each branch. The Ka/Ks values associated with terminal branches between modern species and their most recent reconstructed ancestors were employed in the subsequent analyses. In order to detect selection pressure, Ka/Ks ratio greater than 1, less than 1 and equal to 1 represents positive selection, negative or stabilizing selection and neutral selection, respectively.

### RNA-seq data analysis of NBS-encoding genes

For expression profiling of NBS-encoding genes, we used RNA-seq data that was generated earlier and submitted into GEO database. Transcript abundance is calculated by fragments per kilobase of exon model per million mapped reads (FPKM) and the FPKM values were log2 transformed. A hierarchical cluster was created using the Cluster 3.0 and heat map generated using TreeView Version 1.60 software [41].

## Results

### Identification and classification of NBS genes in *A. thaliana*and *Brassica*species

Although, previously NBS-encoding R genes in *A. thaliana* and *B. rapa* were described by Meyers et al. [10] and Mun et al. [42] respectively, but their analysis were based on old version of TAIR in *A. thaliana* and incomplete genome sequences in *B. rapa*. In the genome assemblies of *B. oleracea*, *B. rapa* and *A. thaliana*, 157, 206 and 167 NBS-encoding genes respectively were identified using the HMM profile from the Pfam database [30]. According to gene structure and protein motifs, we categorized these putative NBS-encoding genes into seven different classes: TNL (40, 93 and 79 for *B. oleracea*, *B. rapa* and *A. thaliana*, respectively), TIR-NBS (29, 23 and 17), CNL (6, 19 and 17), CC-NBS (5, 15 and 8), NBS-LRR (24, 27 and 20) and NBS (53, 29 and 26) (Table 1, Additional file 1: Table S1). We employed HMM search to identify genes with open reading frames that encode TIR domain based on whole genomes of sequenced plant species. By excluding genes that contain NBS domains, we obtained the genes that encode only TIR domain (TIR-X type genes). Although, the number of NBS-encoding genes in *B. oleracea* is less than that of *A. thaliana* and *B. rapa* but genes with truncated domains of NBS, TIR-NBS and TIR-X are more than these species. The total number of NBS-encoding genes in these three species is very close regardless of genome size and WGD/WGT, suggesting WGT might not result in more R genes in *Brassica* species. Much more TNL type genes than CNL ones, and more TIR-NBS than CC-NBS were also observed in these three species.

**Table 1:** **Statistics of predicted NBS-encoding genes in sequenced plant species**

Categories		Bo	Br	At	Tc	Pt	Vv	Mt
**NBS-LRR type**	**TIR-NBS-LRR**	40	93	79	8	78	97	118
	**CC-NBS-LRR**	6	19	17	82	120	203	152
	**NBS-LRR**	24	27	20	104	132	159	-
**NBS type**	**TIR-NBS**	29	23	17	4	10	14	38
	**CC-NBS**	5	15	8	46	14	26	25
	**NBS**	53	29	26	53	62	36	328
**Total NBS**		157	206	167	297	416	535	661
**Total TIR-NBS**		69	116	96	12	88	111	156
**Total CC-NBS**		11	34	25	128	134	229	177
TIR-X*		82	42	46	17	67	10	92
**Total**		239	248	213	314	483	545	753

Note: *Bo-B. oleracea; Br-B. rapa; At-A. thaliana; Tc-T. cacao, Pt-P. trichocarpa, Vv-V. vinifera; Mt- M. truncatula*

*identified in present study.

### Genomic distribution on chromosomes/pseudomolecular chromosomes

**Figure 1:**
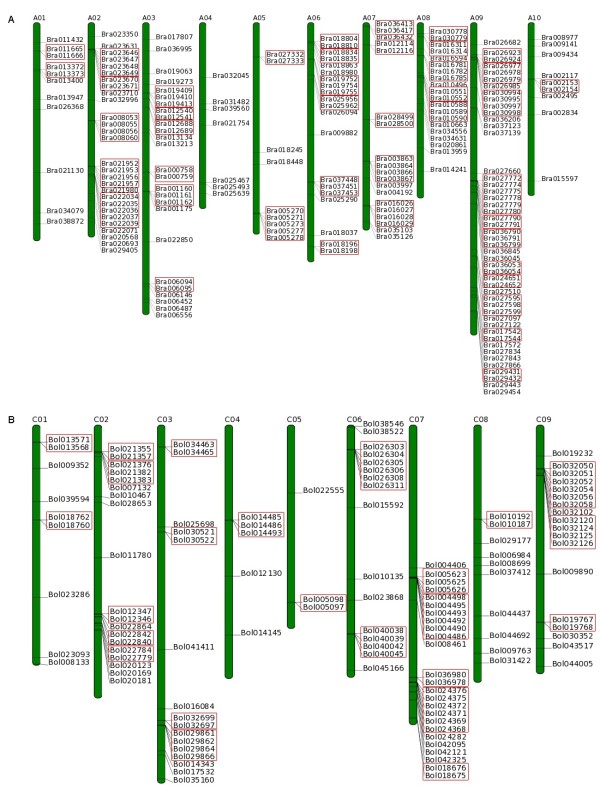
**NBS-encoding genes and corresponding clusters distribution of NBS-encoding genes in** ***B. rapa*** **and** ***B. oleracea*** **genomes. A.** A01 ~ A10 represent pseudo-chromosomes of *B. rapa* genome. **B.** C01 ~ C09 represent pseudo-chromosomes of *B. oleracea* genome. Green bars represent pseudo-chromosomes. Black line on green bars stands for the location of NBS-encoding genes on pseudo-chromosomes. Colorful boxes stand for clusters of NBS-encoding genes in corresponding genomes.

NBS-encoding genes for the three species were mapped onto pseudo-molecules/ chromosomes [121 (77.1%) genes in *B. oleracea*, 197 (95.6%) genes in *B. rapa* and 167 (100%) genes in *A. thaliana*] and the rest [36 (22.9%) genes in *B. oleracea* and 9 (4.4%) genes in *B. rapa*] were located on the unanchored scaffolds (Figure 1). The distribution of these genes is uneven: some chromosomes (e. g. C07 in *B. oleracea* representing the 20.7% of the NBS-encoding genes) have more genes and the rest chromosomes have fewer genes (e. g. C05 in *B. oleracea*), and many of these genes reside in a cluster manner. R genes existing in clusters may facilitate the evolutionary process through producing novel resistance genes via genome duplication, tandem duplication and gene recombination [43]. According to the cluster defined by Richly et al. [44] and Meyers et al. [10] as two or more genes falling within eight ORFs, we found that the percentage of NBS genes on chromosomes in clusters in *B. oleracea* (60.3%) and *A. thaliana* (61.7%) is higher than that of *B. rapa* (59.4%). In *B. oleracea*, 73 NBS genes, representing 60.3% of total genes on chromosomes, were located in 24 clusters and the remaining 48 genes were singletons. Five clusters containing 19 NBS genes were identified on the chromosome C07 (Figure 1A). The *B. rapa* genome carries 117 (59.4%) NBS genes with TIR domain and CC motif in 43 clusters and remaining 80 genes were found as singletons on chromosomes. Among the 43 clusters, 11 with 31 genes were located on chromosome A09 (Figure 1B). In *A. thaliana*, 103 (61.7%) NBS genes with TIR domain and CC motif were mapped in 37 clusters whereas the remaining 64 genes were found as singletons. The numbers of genes in clusters ranged from two to six in both *Brassica* species and two to nine in *A. thaliana*.

Further, more numbers of homogenous clusters was observed in *B. rapa* and *A. thaliana* than *B. oleracea*. In *B. oleracea* among 24 identified clusters, 5 were homogenous and one of them containing four genes (Bol040038, Bol040039, Bol040042, and Bol040045) with TN domain configuration was located on chromosome C06. Most of the clusters (18) are heterogenous with distantly related NBS domains. Fifteen clusters in each of *B. rapa* and *A. thaliana* were found to be homogenous containing the NBS-encoding genes mostly from TNL domain combination.

### Phylogenetic analysis of NBS-encoding genes in *B. oleracea*, *B. rapa*and *A. thaliana*

Comparative phylogenetic relationship of NBS-encoding genes in *B. oleracea*, *A. thaliana* and *B. rapa* represents two major groups of TNL (348 genes) and CNL (138 genes) containing genes from three species. In composite phylogenetic tree, TNL and CNL groups were further divided into three sub-groups, TNL-I-III and CNL-I-III (Additional file 2: Figure S1). We did not observe any strict grouping of N, NN and NL domain containing proteins and these kinds of proteins were clustered in both TNL and CNL groups. From phylogenetic tree, we can differentiate that the number of NBS-encoding genes for three species in each subgroup was not identical. In TNL group all sub-trees comprised genes with full length TIR-NBS-LRR ORFs, truncated and complex domains. TNL-I subgroup was found to be the largest one containing 245 NBS members in total and greater part in this subgroup was from *B. rapa* (106 NBS members). This subgroup included the largest part of the full length TNLs and second and third prevalent classes are TN and N type genes respectively. The domain arrangement was found to be highly diverse and NBS-encoding genes from three species with thirteen different complex and unusual domain combinations of TNNL, TCNL, TNTN, TNLT, TNNTNNL, NLTNL, NNL, TNLTNL, CTN, TNN, TTN, TNLN and LTNL were identified in this subgroup. In subgroup TNL-II, more than half of the genes were from *B. oleracea* and others were from *B.rapa* and *A. thaliana.* This subgroup along with various complex domain arrangement containing genes also carried most of the full length TNLs. TNL-III was the smallest subgroup with majority of genes from *B. oleracea* (5 genes) and a single gene from each of B*. rapa* and *A. thaliana. B. oleracea* gene, Bol044437 with unusual domain arrangement TNNL also clustered in this subgroup.

CNL group was further divided into three distinct subgroups represented by genes from all the three species and we also observed one CNL subgroup which was already recognized in *A. thaliana*. However, CNL group is not much variant and only few complex domain arrangements are evident; NNL, CNNL and CNNN. In CNL-1 subgroup, out of 5 clustered *A. thaliana* genes, 4 genes (AT4G33300.1, AT1G33560.1, AT5G04720.1 and AT5G47280.1) were also grouped in the respective *A. thaliana* CNL-A subgroup as identified and described by Meyers et al. 2003. Both CNL-II and CNL-III subgroups included most of NBS-encoding genes from *B. rapa* and *A. thaliana* and fewer genes from *B. oleracea* species*.* NBS-encoding genes with N and CN type truncated domains were observed more in CNL-II subgroup and one *B. rapa* gene (Bra037453) with unusual domain, CNNN also clustered here. Subgroup CNL-III was represented by 73 genes and most of the members (36) were full length CNL ORFs. Four *B. rapa* genes (Bra030779, Bra027097, Bra019752, Bra015597) with unusual domains NNL and CNNL were also identified in this subgroup.

### Expression analysis of NBS-encoding genes in different tissues

To investigate the expression pattern of NBS-encoding genes, we compared the transcript abundance in different tissues using RNA-seq data from GEO database. The expression profile of NBS-encoding genes in *B. oleracea* could be classified into two major groups (Bol-A and Bol-B) (Additional file 3: Figure S2A). Eighty eight genes belonging to Group Bol-A, further divided into two subgroups, Bol-A1 and Bol-A2. In *B. oleracea* in subgroup Bol-A1, three genes (Bol017532, Bol029866 and Bol013571) expressed relatively higher in root and stalk indicating their tissue-specific role in these tissues. Majority of genes in subgroup Bol-A2 were found to be upregulated in root and callus (for example, Bol038522 displayed more expression in root and callus and Bol024369 was abundant only in root tissue) but down regulated in stalk, leaf, flower and silique. Up regulation of these genes in callus suggests their induction under wounding. However, eighteen genes in group Bol-B displayed differential expression in different tissues and among all the genes in this subgroup, Bol009890 exhibited highest expression in leaf and Bol036980 showed more transcript level in flower tissue.

In *B. rapa*, genes could be categorized into two main groups, Bra-A and Bra-B (Additional file 3: Figure S2B). The Bra-A group was further classified into Bra-A1 (74 genes), Bra-A2 (45 genes) and Bra-A3 (28 genes). In subgroup Bra-A1 of B. *rapa,* most of genes displayed high transcript accumulation in root, stalk and callus which indicates that they may expression pattern differentially. Among the other genes, Bra006146 showed high expression in vegetative tissue (root, stalk and leaf) and Bra004192 and Bra035103 highly expressed in stalk and leaf. In subgroup Bra-A2, where a number of genes were expressed more in root and callus. However, Bra018810 displayed highest expression in silique suggesting its silique-specific role. In Subgroup Bra-A3, some genes showed the preferential transcript level in stalk and flower and some genes relatively expressed higher in flower, silique and callus. For example, Bra008055 accumulated more transcripts in leaf, flower and callus, Bra008056 in flower and Bra026094 in stalk and silique. Most of genes in group Bra-B showed high expression in stalk and leaf as compared to other tissues and Bra009882, Bra008053, Bra018834, Bra027866, Bra026368 and Bra030778 highly expressed in leaf tissues. This may specify that genes in this subgroup act as positive regulator in leaf tissues.

Taken together, we suggest that NBS-encoding genes exhibited differential expression pattern in different tissues and several genes are induced by wounding in *B. oleracea* and *B. rapa* genomes. Some NBS-encoding genes showed higher expression in same tissue indicating their functional conservation, but others were more abundant in different tissues which point toward their functional differences. According to expression pattern of NBS-encoding genes in different tissues, it would be interesting to functionally characterize these genes for pathogen defense response, especially race- and species-specific pathogens in Brassica species*.*

### Whole genome duplication analysis of NBS-encoding genes

**Figure 2:**
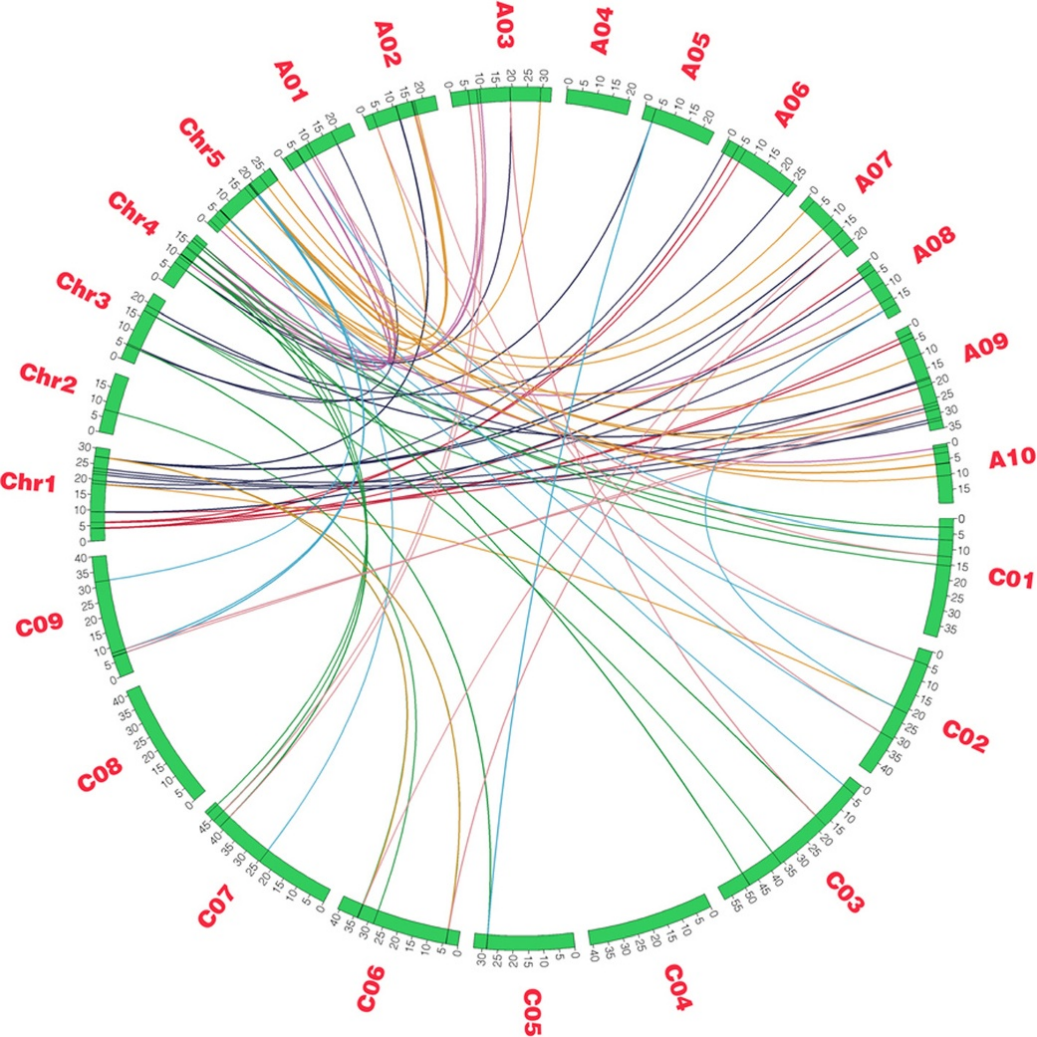
**Syntenic relationship of NBS-encoding genes between** ***A. thaliana*** **and** ***Brassica*** **genomes.** Green bars represent chromosomes of three species. A01 ~ A10 represent pseudo-chromosomes of *B. rapa* genome, C01 ~ C09 represent pseudo-chromosomes of *B. oleracea* genome and Chr1 ~ Chr5 represent chromosomes of *A. thaliana* genome. Black line on green bars stands for the location of NBS-encoding genes on chromosomes/pseudo-chromosomes. Colorful lines stand for the relationship of orthologous gene pairs between different species.

*A. thaliana* genome has experienced two recent whole genome duplication (named α and β) within the crucifer (*Brassicaceae*) lineage and one triplication event (γ) that is probably shared by most dicots (asterids and rosids) [45]. The ancestor of diploid *Brassica* species and *A. thaliana* lineages diverged about 20 MYA and subsequently a whole genome triplication (WGT) event occurred in the *Brassica* ancestor approximately 16 MYA. As WGT of the *Brassica* ancestor, NBS-encoding genes in the *A. thaliana* genome might have triplicated orthologous copies in *B. rapa* and *B. oleracea*. Since, *A. thaliana* is considered a model plant system for plant molecular biology research and most of its genes have been functionally characterized. Therefore, we traced these orthologous gene pairs between *A. thaliana* and *Brassica* species to detect the NBS-encoding genes in evolutionary history. From analysis of orthologous regions for genome-wide comparative analysis, we obtained 42 orthologous gene pairs between *A. thaliana* and *B. oleracea*, 62 between *A. thaliana* and *B. rapa* and 24 between *B. oleracea* and *B. rapa*, which are shown in Figure 2 developed by Circos software [46] (Figure 2).

Out of 42 gene pairs between *A. thaliana* and *B. oleracea*, 26 *A. thaliana* NBS genes were shown to retain one copy, 5 *A. thaliana* NBS genes retained two copies and only 2 genes corresponding to AT4G19500.1 and AT4G19510.1 each preserved tripled copies after triplication in *B. oleracea*. In total, 42 NBS genes in *B. oleracea* genome have 33 corresponding genes in *A. thaliana* genome. *A. thaliana* corresponding genes in *B. oleracea* were located on different chromosomes and some gene pairs (which retained single copy in *B. oleracea*) and 3 out of 5 *A. thaliana* corresponding genes (which retained two copies in *B. oleracea*) preserved domain structure (Table 2).

**Table 2:** **Orthologous gene pairs of NBS-encoding genes between** ***A. thaliana*** **and** ***B. oleracea*** **genomes**

***A. thaliana***	NBS-encoding genes in ***A. thaliana***				***B. oleracea***	NBS-encoding genes in ***B. oleracea***			
	Gene_Type	Location	ORF Length	No. of exons		Gene_Type	Location	ORF Length	No. of exons
AT1G27170.1	TIR-NBS-LRR	Chr1	4,858	5	Bol037684	TIR-NBS-LRR	NY	4,783	5
AT1G50180.1	NBS	Chr1	2,901	5	Bol011780	CC-NBS	C02	3,507	3
AT1G63730.1	TIR-NBS-LRR	Chr1	3,362	4	Bol022619	NBS-LRR	NY	4,656	3
AT1G72870.1	TIR-NBS	Chr1	2,161	2	Bol026308	TIR-NBS	C06	2,317	2
AT1G72890.1	TIR-NBS	Chr1	1,770	2	Bol026304	NBS	C06	830	2
					Bol040038	TIR-NBS	C06	1,565	3
AT1G72950.1	TIR-NBS	Chr1	1,395	2	Bol026303	TIR-NBS	C06	1,232	2
					Bol040042	TIR-NBS	C06	2,357	3
AT2G17060.1	TIR-NBS-LRR	Chr2	4,466	6	Bol023868	CC-NBS-LRR	C06	2,938	2
AT3G14460.1	NBS-LRR	Chr3	4,274	1	Bol005097	NBS-LRR	C05	3,623	1
AT3G14470.1	NBS-LRR	Chr3	3,307	1	Bol005098	NBS	C05	9,997	3
AT3G46730.1	NBS	Chr3	2,543	1	Bol041411	NBS	C03	164	1
					Bol018762	NBS	C01	869	1
AT3G51560.1	TIR-NBS-LRR	Chr3	4,105	5	Bol010610	TIR-NBS-LRR	NY	5,368	4
AT3G51570.1	TIR-NBS-LRR	Chr3	4,098	5	Bol010611	TIR-NBS	NY	4,246	6
AT4G12010.1	TIR-NBS-LRR	Chr4	4,182	5	Bol008302	TIR-NBS-LRR	NY	5,143	5
					Bol030522	TIR-NBS-LRR	C03	3,222	4
AT4G12020.2	NBS-LRR	Chr4	7,992	16	Bol030521	NBS	C03	4,504	6
AT4G19050.1	NBS-LRR	Chr4	3,684	2	Bol009352	NBS-LRR	C01	3,341	1
AT4G19500.1	TIR-NBS-TIR-NBS-LRR	Chr4	4,736	5	Bol003710	NBS-LRR	NY	4,676	3
					Bol024375	TIR-NBS-LRR	C07	4,087	6
					Bol029862	TIR-NBS-LRR	C03	2,445	4
AT4G19510.1	TIR-NBS-LRR	Chr4	5,316	6	Bol003711	TIR-NBS	NY	3,941	4
					Bol024376	NBS	C07	3,424	4
					Bol029861	TIR-NBS	C03	3,947	5
AT4G19520.1	TIR-NBS-LRR	Chr4	4,421	4	Bol024371	TIR-NBS-LRR	C07	13,901	6
AT4G19530.1	TIR-CC-NBS-LRR	Chr4	5,538	5	Bol024372	TIR-NBS-LRR	C07	6,114	4
AT4G26090.1	CC-NBS-LRR	Chr4	3,534	1	Bol039594	CC-NBS-LRR	C01	2,723	1
AT4G27190.1	CC-NBS-LRR	Chr4	2,957	1	Bol042325	CC-NBS-LRR	C07	3,053	1
AT4G33300.1	NBS-LRR	Chr4	5,475	5	Bol013568	NBS	C01	2,067	5
AT4G36140.1	TIR-NBS-TIR-NBS-LRR	Chr4	5,523	7	Bol018676	NBS-LRR	C07	4,440	7
AT5G04720.1	NBS	Chr5	3,172	5	Bol002454	NBS-LRR	NY	3,733	5
AT5G17880.1	TIR-NBS-LRR	Chr5	4,225	6	Bol019768	TIR-NBS	C09	2,856	5
					Bol034463	TIR-NBS-LRR	C03	2,203	3
AT5G17970.1	TIR-NBS-LRR	Chr5	2,620	4	Bol021382	NBS-NBS	C02	4,845	2
AT5G45200.1	TIR-NBS-LRR	Chr5	6,365	5	Bol032050	TIR-NBS-LRR	C09	9,525	4
AT5G45210.1	TIR-NBS-LRR	Chr5	2,913	4	Bol032051	TIR-NBS	C09	5,857	4
AT5G45240.1	TIR-NBS-LRR	Chr5	5,383	10	Bol005623	NBS	C07	2,409	3
AT5G45250.1	TIR-NBS-LRR	Chr5	4,108	5	Bol032054	TIR-NBS	C09	4,575	6
AT5G45490.1	NBS	Chr5	1,394	1	Bol022842	NBS	C02	728	1
AT5G46450.1	TIR-NBS-LRR	Chr5	3,928	5	Bol032126	NBS-LRR	C09	2,893	3
AT5G46470.1	TIR-NBS-LRR	Chr5	7,040	6	Bol032125	NBS	C09	671	2

Note: NY, not yet assigned to a chromosome.

Out of 62 gene pairs between *A. thaliana* and *B. rapa,* 40 *A. thaliana* NBS genes were shown to retain one copy, 8 *A. thaliana* NBS genes retained two copies and only two genes (AT4G26090.1 and AT1G72890.1) preserved tripled copies in *B. rapa*. At last, we got 50 *A. thaliana* NBS genes compared to 62 NBS genes in *B. rapa* genome. Gene pairs in *B. rapa* corresponding to *A. thaliana* were located on different chromosomes. Further, some genes (which retained single copy in *B. rapa*), 5 out of 8 *A. thaliana* NBS genes (which retained two copies in *B. rapa*) and 2 genes (which retained tripled copies in *B. rapa*) preserved domain configuration in *B. rapa* (Table 3)*.*

**Table 3:** **Orthologous gene pairs of NBS-encoding genes between** ***A. thaliana*** **and** ***B. rapa*** **genomes**

***A. thaliana***	Attribute of NBS-encoding genes in ***A. thaliana***				***B. rapa***	Attribute of NBS-encoding genes in ***B. rapa***			
	Gene_Type	Location	ORF_Length	No. of exons		Gene_Type	Location	ORF_Length	No. of exons
AT1G50180.1	NBS	Chr1	2,901	5	Bra014241	CC-NBS	A08	4,047	3
AT1G58410.1	NBS	Chr1	3,070	3	Bra027866	CC-NBS	A09	2,986	3
AT1G59620.1	NBS	Chr1	3,401	5	Bra035424	CC-NBS	NY	3,017	3
					Bra016781	CC-NBS-LRR	A08	2,662	2
AT1G12290.1	CC-NBS-LRR	Chr1	2,888	1	Bra026979	CC-NBS-LRR	A09	2,744	1
AT4G26090.1	CC-NBS-LRR	Chr4	3,534	1	Bra013947	CC-NBS-LRR	A01	2,723	1
					Bra019063	CC-NBS-LRR	A03	3,029	1
					Bra037139	CC-NBS-LRR	A09	3,023	4
AT3G51560.1	TIR-NBS-LRR	Chr3	4,105	5	Bra036791	TIR-NBS-LRR	A09	10,379	6
AT1G17610.1	NBS	Chr1	1,462	1	Bra030997	NBS	A09	1,262	1
AT1G52660.1	NBS	Chr1	1,321	3	Bra018980	NBS	A06	1,308	3
AT3G15700.1	NBS	Chr3	1,240	2	Bra021130	NBS	A01	1,322	2
AT3G46710.1	NBS	Chr3	2,543	1	Bra018198	NBS	A06	2,078	5
AT4G19060.1	NBS	Chr4	1,384	1	Bra013373	NBS	A01	716	1
AT5G11250.1	TIR-NBS-LRR	Chr5	3,982	4	Bra008977	NBS	A10	518	1
AT5G45490.1	NBS	Chr5	1,394	1	Bra021980	NBS	A02	1,130	1
AT5G56220.1	NBS	Chr5	3,102	1	Bra002834	NBS	A10	2,918	1
AT1G12210.1	NBS-LRR	Chr1	2,657	1	Bra019755	NBS-LRR	A06	2,682	2
AT1G12220.1	NBS-LRR	Chr1	2,882	1	Bra019754	NBS-LRR	A06	2,672	1
					Bra016311	NBS-LRR	A08	4,678	5
AT3G14460.1	NBS-LRR	Chr3	4,274	1	Bra027333	NBS-LRR	A05	4,229	1
AT3G14470.1	NBS-LRR	Chr3	3,307	1	Bra027332	NBS-LRR	A05	3,128	1
AT4G12020.2	NBS-LRR	Chr4	7,992	16	Bra000758	NBS-LRR	A03	4,423	5
AT4G19050.1	NBS-LRR	Chr4	3,684	2	Bra013372	NBS-LRR	A01	3,541	2
AT4G27190.1	CC-NBS-LRR	Chr4	2,957	1	Bra026368	NBS-LRR	A01	2,933	1
AT4G33300.1	NBS-LRR	Chr4	5,475	5	Bra034556	NBS-LRR	A08	3,140	5
AT5G04720.1	NBS	Chr5	3,172	5	Bra009434	NBS-LRR	A10	3,120	5
					Bra022036	NBS-LRR	A02	6,957	6
AT5G66900.1	CC-NBS-LRR	Chr5	3,024	5	Bra012116	NBS-LRR	A07	4,738	7
AT1G61310.1	CC-NBS-LRR	Chr1	2,880	1	Bra027097	NBS-NBS-LRR	A09	2,736	3
AT1G17615.1	TIR-NBS	Chr1	1,226	2	Bra025962	TIR-NBS	A06	1,634	2
AT1G72840.1	TIR-NBS-LRR	Chr1	4,529	4	Bra008053	TIR-NBS	A02	8,376	5
AT1G72860.1	TIR-NBS	Chr1	4,550	3	Bra008056	TIR-NBS	A02	1,832	2
AT1G72890.1	TIR-NBS	Chr1	1,770	2	Bra016029	TIR-NBS	A07	1,428	2
					Bra008060	TIR-NBS	A02	1,685	2
					Bra003864	TIR-NBS	A07	1,661	2
AT1G72950.1	TIR-NBS	Chr1	1,395	2	Bra016028	TIR-NBS	A07	1,366	2
AT5G45240.1	TIR-NBS-LRR	Chr5	5,383	10	Bra021957	TIR-NBS	A02	7,454	2
AT1G27170.1	TIR-NBS-LRR	Chr1	4,858	5	Bra024651	TIR-NBS-LRR	A09	3,671	4
AT1G27180.1	TIR-TIR-NBS	Chr1	6,247	6	Bra016314	TIR-NBS-LRR	A08	4,645	5
AT1G63730.1	TIR-NBS-LRR	Chr1	3,362	4	Bra027791	TIR-NBS-LRR	A09	13,529	6
					Bra003867	TIR-NBS-LRR	A07	6,912	10
AT3G51570.1	TIR-NBS-LRR	Chr3	4,098	5	Bra036790	TIR-NBS-LRR	A09	4,182	6
AT4G12010.1	TIR-NBS-LRR	Chr4	4,182	5	Bra029431	TIR-NBS-LRR	A09	4,646	5
					Bra000759	TIR-NBS-LRR	A03	3,934	5
AT4G16890.1	TIR-NBS-LRR	Chr4	4,949	7	Bra012688	TIR-NBS-LRR	A03	5,918	9
AT4G19500.1	TIR-NBS-TIR-NBS-LRR	Chr4	4,736	5	Bra013400	TIR-NBS-LRR	A01	5,825	8
					Bra012540	TIR-NBS-LRR	A03	4,567	8
AT4G36150.1	TIR-NBS-LRR	Chr4	3,992	5	Bra011666	TIR-NBS-LRR	A01	4,726	6
AT5G17680.1	TIR-NBS-LRR	Chr5	4,154	4	Bra013959	TIR-NBS-LRR	A08	4,066	4
AT5G17970.1	TIR-NBS-LRR	Chr5	2,620	4	Bra002117	TIR-NBS-LRR	A10	3,627	4
					Bra023647	TIR-NBS-LRR	A02	2,888	4
AT5G18350.1	TIR-NBS-LRR	Chr5	4,500	6	Bra002154	TIR-NBS-LRR	A10	4,737	5
					Bra006452	TIR-NBS-LRR	A03	8,631	9
AT5G41550.1	TIR-NBS-LRR	Chr5	3,553	4	Bra028500	TIR-NBS-LRR	A07	4,012	4
AT5G45230.1	TIR-NBS-LRR	Chr5	6,156	6	Bra021956	TIR-NBS-LRR	A02	4,277	5
AT5G45250.1	TIR-NBS-LRR	Chr5	4,108	5	Bra027599	TIR-NBS-LRR	A09	3,931	5
AT5G46450.1	TIR-NBS-LRR	Chr5	3,928	5	Bra017542	TIR-NBS-LRR	A09	3,362	5
AT5G46470.1	TIR-NBS-LRR	Chr5	7,040	6	Bra017544	TIR-NBS-LRR	A09	5,508	7
AT1G17600.1	TIR-NBS-LRR	Chr1	3,322	4	Bra030998	TIR-NBS-LRR-NBS	A09	5,997	8
AT4G36140.1	TIR-NBS-TIR-NBS-LRR	Chr4	5,523	7	Bra011665	TIR-NBS-LRR-TIR	A01	4,843	6
AT5G18370.1	TIR-NBS-LRR	Chr5	3,890	4	Bra002153	TIR-NBS-NBS-LRR	A10	7,583	7

Note: NY, not yet assigned to a chromosome.

The ancestor of *Brassica* species has experienced whole genome triplication and thus provided sufficient genomic materials to study retention and loss of NBS-encoding genes. In order to detect retention or loss of NBS-encoding genes after WGT, we studied the *A. thaliana* NBS genes, which have corresponding genes in *Brassica* species. There are 33 *A. thaliana* NBS genes compared to 42 *B. oleracea* NBS genes and 50 *A. thaliana* NBS genes compared to 62 *B. rapa* NBS genes, which have 24 overlapping NBS genes. In other words, 59 NBS genes in *A. thaliana* genome were identified on triplicated regions and generated triple copies in *Brassica* species, representing 35.32% of total NBS genes in *A. thaliana* genome. Because of evolutionary constraints, 42 NBS genes were retained on triplicated regions, representing 26.75% of total NBS genes in *B. oleracea* genome and 62 NBS genes were retained on triplicated blocks, which represent 30.1% of whole NBS genes in *B. rapa* genome.

### Tandem duplication analysis of NBS-encoding genes

Whole genome and/or tandem duplication is thought to be source of complexity and diversity for plant species and allow them to adapt to the changed environmental conditions. In *B. oleracea* genome, 68 of 157 identified NBS-encoding genes, representing 43.3% genes were formed by tandem duplication and distributed in 26 tandem arrays of 2–6 genes. The chromosome map identified 21 tandem arrays including 57 NBS-encoding genes unevenly distributed on seven of the nine chromosomes and remaining 11 genes were unanchored on scaffold sequences. Genes with CNL or CN domain were not appeared in tandem arrays. Single tandem duplicated array containing two genes were identified on each of chromosome C01 and C05 with N and NL domains. Each of the chromosomes C02 and C03 carried four tandem arrays with 2–4 genes. The chromosome C06 (2–5 genes in arrays) and C09 (2–4 genes in arrays) carried two and three tandem arrays respectively. The highest number of tandem arrays (6) with 17 genes was found on chromosome C07 which contains the highest number of R genes in the genome. In *A. thaliana* genome, out of 167 NBS genes 93 (55.7%) genes were tandemly duplicated and positioned on chromosomes in 37 tandem arrays. The tandem duplicated genes were distributed in tandem arrays of 2–6 genes. In *B. rapa* genome, 97 genes (47.1%) were tandemly duplicated and 93 genes were located on chromosomes in 38 tandem arrays while two tandem arrays were located on scaffold sequences. The number of duplicated genes range from 2–5 genes in tandem arrays (Table 4, Additional file 4: Table S2).

**Table 4:** **Statistics of tandem arrays for NBS-encoding genes in** ***A. thaliana*** **,** ***B. rapa*** **and** ***B. oleracea***

Categories	Total NBS genes	Tandem genes	Percentage (%)	Tandem arrays	Common tandem genes	Common tandem arrays	Located on chromosomes	Unanchored
***A. thaliana***	167	93	55.7	37	20	10	93	/
***B. rapa***	206	97	47.1	40	14	7	93	4
***B. oleracea***	157	68	43.3	26	18	9	57	11

In order to detect the fate of tandem arrays in *Brassica* lineage after split from *Arabidopsis thaliana*, we investigated the orthologous gene pairs in tandem array among *B. oleracea*, *B. rapa* and *A. thaliana* genomes. 10 two-gene tandem arrays of *A. thaliana* have corresponding two-gene tandem arrays in *B. oleracea* and *B. rapa* genomes, and further 7 and 9 two-gene tandem arrays have retained their copies in *B. rapa* and *B. oleracea* genome, respectively (Additional file 5: Table S3). Out of 10 two-gene tandem arrays in *A. thaliana*, 4 *A. thaliana* two-gene tandem arrays were co-retained tandem arrays and have corresponding two-gene tandem arrays in *B. rapa* and *B. oleracea* genome, 3 two-gene tandem arrays have retained in *B. rapa* genome and 3 two-gene tandem arrays have retained in *B. oleracea* genome. Among 157 NBS-encoding genes in *B. oleracea*, 68 genes were tandem duplicated genes. 18 of 68 genes were conserved and have ancient copies, indicating that those 18 genes were generated before divergence of *A. thaliana* and *Brassica* ancestor. Consequently, 50 NBS-encoding genes were distributed in species-specific tandem arrays in *B. oleracea* genome. In *B. rapa* genome, 97 tandem duplicated genes representing 47.1% of 206 NBS-encoding genes in total, contained 14 genes belonging to tandem of pre-split. 83 genes were species-specific tandem duplicated genes in *B. rapa* genome. There are 93 genes identified as tandem duplicated genes in *A. thaliana* genome and 20 tandem duplicated genes are pre-split tandem genes, named common tandem duplicated genes, which were generated before divergence of *A. thaliana* and *Brassica* ancestor. Out of 20 common tandem genes, 8 genes retained copies in *Brassica* species and those corresponding co-retained tandem genes were race-specific tandem duplicated genes in *Brassica* species.

### Syntenic analysis of orthologous gene pairs for NBS-encoding genes among *B. oleracea*, *B. rapa*and *A. thaliana*

**Figure 3:**
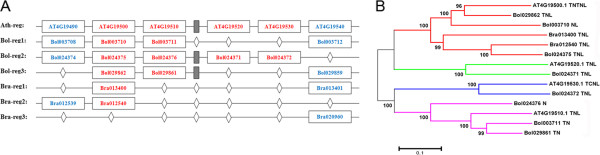
**Correspondence and phylogenetic relationship of orthologous gene pairs for NBS-encoding genes among** ***B. oleracea*** **,** ***A. thaliana*** **and** ***B. rapa*** **. A.** corresponding relationship of orthologous gene pairs for NBS genes among three species. Ath-reg represents target region on *A. thaliana* Chr4 that a tandem array of NBS genes located. Bol-reg1 ~ 3 and Bra 1 ~ 3 represent trplicated regions of target region in *B. oleracea* and *B. rapa* genome, respectively. Blue characters in rectangle stands for non-R genes and red character in rectangle stands for NBS genes. Diamond stands for gene absence at the locus on these chromosome regions. Gray solid rectangle stands for non-R genes within the tandem array. **B.** phylogenetic relationship of orthologous gene pairs for NBS genes among *B. oleracea*, *A. thaliana* and *B. rapa*. different colors can distinguish different sub-trees.

Whether retention of *Brassica* triplets is random or determined by their genomic position or function remains unknown. We investigated the syntenic relationship of sample region in *A. thaliana* containing four genes compared to syntenic counterpart regions in *B. oleracea* and *B. rapa* genomes to detect deletion or loss on triplicated regions among 3 species. The genes from AT4G19500 ~ AT4G19530 were found in tandem arrays located on the sample region of chromosome 4 in *A. thaliana* genome. Only two genes in this tandem array (AT4G19500 and AT4G19510) preserved tripled copies and other two genes (AT4G19520 and AT4G19530) have retained one copy in *B. oleracea* genome respectively. In *B. rapa* genome, we found that only AT4G19500 gene preserved two copies and other members of this tandem arrays were missed or deleted (Figure 3A). From analysis of orthologous gene pairs, it is clear that this region is three copied region retained in *B. oleracea* genome and two copied regions in *B. rapa* genome. As to every member of tandem array in *A. thaliana* has a corresponding copy on triplicated regions of *B. oleracea* and also has a clear syntenic relationship between two species, we can speculate that this tandem array was generated before the split of *A. thaliana* and *Brassica* ancestor.

From phylogenetic analysis, it is clear that AT4G19500.1 have three homologous genes (Bol029862, Bol003710 and Bol024375) in *B. oleracea* and two homologous genes (Bra013400 and Bra012540) in *B. rapa*, which were clustered in one phylogenetic sub tree corresponding to syntenic relationship. The second member of tandem array, AT4G19510.1 have three homologous genes (Bol024376, Bol003711 and Bol029861) only in *B. oleracea* and did not retain any copy in *B. rapa* genome, indicating syntenic relationship between two species. Each of tandem array member, AT4G19520.1 and AT4G19530.1 have one homologous genes (Bol024371 and Bol024372) only in *B. oleracea* respectively, which appeared in one phylogenetic sub tree (Figure 3B).

The genes AT4G19500 and AT4G19510 in tandem array might have important role in process of *A. thaliana* diseases resistance, so they retained three copies after triplication in *B. oleracea* genome. AT4G19520 and AT4G19530 might have subjected to less evolutionary pressure leading to other two other two duplicated copies lost in *B. oleracea* genome. We hypothesize that these homologous genes of *B. oleracea* might be resistant to species-specific pathogens or diseases in *B. oleracea* genome. After WGT of *Brassica* ancestor, genomic components were triplicated and redundance data was generated. From evolutionary pressure or environment selection, critical components were retained and others were deleted or lost.

### Expression analysis of orthologous and paralogous gene pairs for NBS-encoding genes among *B. oleracea*, *B. rapa*and *A. thaliana*

Differential expression level of orthologous and paralogous gene pairs for NBS-encoding genes can reflect expression pattern divergence of orthologous and paralogous genes after WGT. Through syntenic analysis among 3 species, we focused on transcript expression level of 5 CNL and 16 TNL NBS-encoding genes in different tissues in *A. thaliana* which have their corresponding orthologous and paralogous genes in *B. rapa* and *B. oleracea* genomes to investigate expression pattern divergence among 3 species.

**Figure 4:**
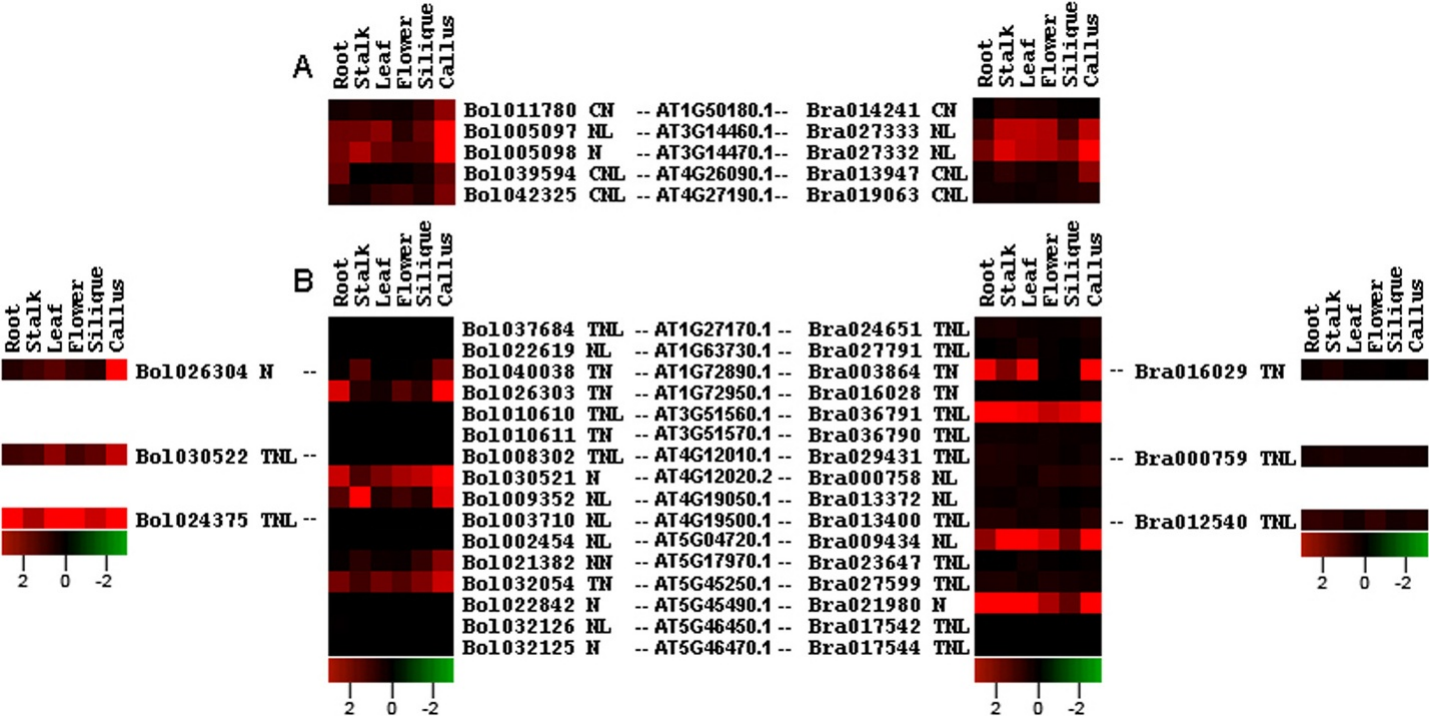
**Heat map representation of orthologous gene pairs for CNL and TNL types between** ***A. thaliana*** **compared to** ***B. oleracea*** **and** ***A. thaliana*** **compared to** ***B. rapa*** **genomes. A**. Heat map representation of orthologous gene pairs for CNL types between *A. thaliana* compared to *B. oleracea* and *A. thaliana* compared to *B. rapa* genomes. **B**. Heat map representation of orthologous gene pairs for TNL types between *A. thaliana* compared to *B. oleracea* and *A. thaliana* compared to *B. rapa* genomes. The tissues used for expression profiling are indicated at the top of each column. The genes are on right or left of expression bar. Color scale bar at the bottom of each heat map represents log2 transformed FPKM values, thereby values 2, 0 and -2 represent positive, zero and negative expression, respectively.

In CNL group in case of orthologs, the expression of two orthologous genes (corresponding to *A. thaliana* gene AT1G50180.1), one in *B. oleracea* (Bol011780) and one in *B. rapa* (Bra014241), was found to be different across the different tissues. Bol011780 showed reduced expression in stalk, silique and moderately expressed in callus, on the other hand we only observed the reduced expression of Bra014241 in stalk, leaf and flower. Orthologous gene Bol039594 in *B. oleracea* (corresponding to *A. thaliana* gene AT4G26090.1) expressed only in root, silique and callus while the expression of its corresponding analogue Bra013947 in *B. rapa* was confined to stalk and callus. Further, Bol042325 (corresponding to *A. thaliana* gene AT4G27190.1) expressed in leaf, flower and callus but expression of its orthologous gene in *B. rapa* (Bra019063) was significantly decreased in all tissues. Another orthologous gene in *B. oleracea,* Bol005097 (corresponding to *A. thaliana* gene AT3G14460.1) was abundantly expressed in leaf, callus and moderately expressed in root, stalk and silique and its orthologous gene, Bra027333 in *B. rapa* displayed high expression in stalk, leaf, flower, callus and reduced expression in root and silique. Bol005098 in *B. oleracea* (corresponding to *A. thaliana* gene AT3G14470.1) abundantly expressed in root, stalk, leaf, callus and exhibited reduced expression in flower and silique, whereas its orthologous gene Bra027332 in *B. rapa*, was ubiquitously expressed in all tissues (Figure 4A). In CNL group, the above mentioned two genes in *A. thaliana* (AT3G14460.1 and AT3G14470.1) are also located in a tandem array and two paralogs in each of *B. oleracea* (Bol005097 and Bol 005098) and *B. rapa* (Bra027332 and Bra027333) were generated by their tandem duplication. When we compared the expression profile between these two paralogs (Bol005097 and Bol 005098) in *B. oleracea*, we found that there was a clear difference in expression level of these two paralogs in different tissues except the root and callus where they transcribe almost at the same level. In *B. rapa*, the expression of Bra027332 and Bra027333 paralogs was significantly high in stalk, leaf, flower and callus, but Bra027332 exhibited moderate and Bra027333 showed low expression level in root and siliques (Figure 4A).

In TNL group in case of orthologs, four orthologs (two in each of *B. oleracea* and *B. rapa*) corresponding to *A. thaliana* gene AT1G72890.1 have been identified. In *B. oleracea* Bol026304 was observed to express in stalk, leaf, flower and callus whereas Bol040038 only expressed in stalk and callus. In *B. rapa* one of the retained orthologous copy (Bra003864) expressed in vegetative tissues and other orthologous gene (Bra016029) was down regulated in all tissues. Another *A. thaliana* gene (AT1G72950.1) retained a single ortholog in each of *B. oleracea* (Bol026303) and *B. rapa* (Bra016028), where Bol026303 was noticed in all tissues but specifically highly expressed in root and callus, while its orthologous gene Bra016028 transcribe at too low level to be detected. In one more case in TNL type, *A. thaliana* gene AT4G12010.1 retained corresponding two orthologous genes in each of *B. oleracea* (Bol008302 and Bol030522) and *B. rapa* (Bra029431 and Bra000759). One of the ortholog Bol030522 expressed more or less in all tissues while rest of the orthologs transcribed at too low level. Furthermore, a single copy in each of *B. oleracea* (Bol030521) and *B. rapa* (Bra000758) was retained corresponding to *A. thaliana* gene AT4G12020.2. Bol030521 ubiquitously expressed in most of the tissues but the expression level of its ortholog, Bra000758 in *B. rapa* was very reduced in all tissues (Figure 4B). In TNL group, a tandem array (AT4G12010.1 and AT4G12020.2) in *A. thaliana* gave rise to three paralogs (two generated by tandem duplication and one by genome triplication) in each of *B. oleracea* (Bol030521, Bol030522 and Bol008302) and *B. rapa* (Bra000758, Bra000759 and Bra029431). Through expression profile comparison it is clear that in *B. oleracea*, Bol030521 highly expressed in root, leaf, flower, silique and callus, Bol030522 distinctly expressed in leaf, silique and callus while the expression level of Bol008302 was very low across all tissues studied. In *B. rapa*, the expression of two paralogs (Bra000758 and Bra029431) was significantly reduced in all tissues while Bra000759 was detected at very low level. In addition to that the other two genes in *A. thaliana*, AT1G72890.1 and AT1G72950.1 have also generated three paralogs (again two generated by tandem duplication and one by genome triplication) in *B. oleracea* and *B. rapa.* In *B. oleracea,* Bol026303 was noticeably expressed in root, flower and callus, Bol026304 showed clear expression only in stalk, leaf and callus, whereas the third paralog Bol040038 was only detected in stalk and callus. In *B. rapa*, Bra003864 showed significantly wide expression in root, stalk, leaf and callus whereas the other two paralogs Bra016028 and Bra016029 exhibited very low expression level in all tissues (Figure 4B).

Through expression divergence analysis in CNL and TNL type by comparing the difference between paralogous and orthologous gene pairs, the results indicate the functional variability of these retained orthologous and paralogous gene copies in *B. oleracea* and *B. rapa*. The expression profile diverged more in paralogous than orthologous gene pairs, consequently paralogous genes might contribute more towards functional divergence than orthologous genes over evolutionary history in Brassicaceae family.

### Comparative evolutionary analyses of orthologous gene pairs for NBS-encoding genes

**Figure 5:**
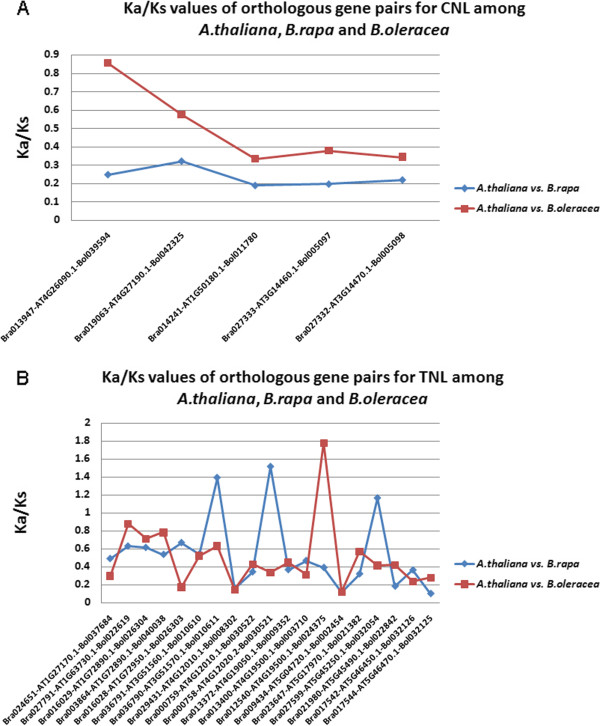
**Comparative analysis of Ka, Ks and Ka/Ks values for CNL and TNL types NBS-encoding gene pairs between** ***A. thaliana*** **compared to** ***B. oleracea*** **and** ***A. thaliana*** **compared to** ***B. rapa*** **genomes.** x**-**axis and y-axis represent values and orthologous gene pair names in *B. oleracea* and *B. rapa* compared to *A. thaliana* separately. **A**. Ka/Ks values of orthologous gene pairs for CNL group; **B**. Ka/Ks values of orthologous gene pairs for TNL group.

Ka/Ks value is the ratio between the number of nonsynonymous substitutions per nonsynonymous site (Ka) and the number of synonymous substitution per synonymous site (Ks). The ratios of the rates of nonsynonymous to synonymous substitutions (Ka/Ks) of orthologous gene pairs were estimated for each branch of the phylogenetic tree using PAML software [40]. The Ka/Ks ratio for orthologous gene pairs was employed to detect the evolutionary selection pattern of NBS-encoding genes among the three species. Through comparative analysis of orthologous gene pairs for NBS-encoding genes among three species, we found 5 CNL and 16 TNL R genes in *A. thaliana* which have their corresponding gene copies in *A. thaliana* compared to *B. rapa* and *A. thaliana* compared to *B. oleracea* genomes. Out of 16 TNL R genes in *A. thaliana*, three genes retained two copies. Thus, we obtained 19 orthologous gene pairs in *A. thaliana* compared to *B. rapa* and *A. thaliana* compared to *B. oleracea* genomes (Figure 5). For CNL type, the mean Ka/Ks ratios of all orthologous genes was 0.497 in *B. oleracea* genome compared to its recent reconstructed ancestor, which is greater (52.72%) than that (0.235) of *B. rapa* genome. The Ka/Ks values of orthologous gene pairs of CNL type R genes were found to show significant differences in *B. oleracea* and *B. rapa* species (P = 0.009 < 0.05, Mann–Whitney U-test, Figure 5A) [47]. The mean Ka/Ks values of all orthologous gene pairs for TNL group was 0.497 in *B. oleracea* genome, which is slightly lower than Ka/Ks value of 0.54 in *B. rapa* genome, but there are no significant differences between the two species (P = 0.759 > 0.05, Mann–Whitney U-test, Figure 5B).

Comparing the mean Ka/Ks ratios for CNL group, we observed that orthologous genes of CNL type NBS-encoding genes in *B. rapa* species experienced stronger pressure about negative selection than *B. oleracea* species which specify that *B. oleracea* species experienced stronger evolutionary constraints than *B. rapa* species in CNL type NBS-encoding orthologous genes. But for TNL group, we could not find significant differences between the two species about the orthologous gene pairs, although the mean Ka/Ks values of all orthologous gene pairs for TNL group in *B. rapa* genome was slightly higher than that of *B. oleracea* genome.

### Analysis of R genes with known functions for specific pathogens in *A. thaliana*and *Brassica*species

We retrieved the characterized gene-for-gene type NBS proteins in *A. thaliana* through PRGdb (http://prgdb.crg.eu/wiki) database [4] and their corresponding genes were obtained from TAIR10 [27]. These pathogen resistant genes were compared with conserved orthologous gene pairs in *Brassica* species and totally four NBS disease resistance genes were obtained in *A. thaliana* which retained the orthologous gene pairs in *Brassica* species. In *A. thaliana*, *RPM1* gene enables dual specificity resistance to two avirulence products (*AvrRpm1* or *AvrB* type III effector protein) of *Pseudomonas syringae*, a causal agent of bacterial blight disease [48]. In the conserved orthologous gene pairs, we found one gene with NBS domain in *B. oleracea* (Bol008133) analogous to the *RPM1*. We also recognized one conserved gene, Bra040356 in *B. rapa* corresponding to *RPM1* but its domain type could not identified. Another disease resistance gene, *RPS2* in *A. thaliana* is known to have NBS, LRR and LZ domains and confers resistance to *P. syringae* avirulence gene *avrRpt2*[49]. Earlier Wroblewski et al. [50] identified *RPS2* homologs with high nucleotide similarity in *Brassica* species, like *B. montana* AF180358 (98% identity), *B. oleracea* AF180357 (98% identity) and *B. rapa* AF180359 (95% identity). Later Malvas et al. [51] also described the involvement of *B. oleracea RPS2* homolog in disease resistance which is constitutively expressed. In our study, two CNL type NBS genes, one in *B. rapa* (Bra013947-previous GenBank ID AF180359) and one in *B. oleracea* (Bol039594- previous GenBank ID AF180357) were found to be conserved parallel to the *RPS2* gene with 94.59% nucleotide similarity. In addition to that both *B. oleracea* and *B. rapa* have retained one copy of (Bra027599) and (Bol032054) genes parallel to *RPS4* which was previously recognized to specify resistance to avirulence gene *avrRps4* from *P. syringae* pv. *Pisi* in *A. thaliana*[52]. Two genes only existed in *B. rapa* (Bra019754 and Bra016781) were preserved with *RPS5* disease resistance gene in *A. thaliana* that is responsible for recognizing avirulence gene *avrPphB* from *P. syringae* pv.*Phaseolicola*[53].

## Discussion

### TNL, TN and TX genes in *Brassica*species

The present study suggests the abundance of TNL class in *Brassicaceae* family (*A. thaliana, B. oleracea* and *B. rapa)* however, comparisons across plant species demonstrated that in *T. cacao, P. trichocarpa, V. vinifera; and M. truncatula* the CNL class predominates. In contrast to the NBS-encoding genes, the current analysis revealed a higher number of TIR-X (TX-lacking both NBS and LRRs) and TIR-NBS (TN-lacking LRRs) genes in *B. oleracea* (82 and 29) than *A. thaliana* (46 and17), *B. rapa* (42 and 23), *Theobroma cacao* (17 and 4), *Populus trichocarpa* (67 and 10) and *Vitis vinifera* (10 and 14). Furthermore, in three *Brassica* species (*A. thaliana, B. oleracea* and *B. rapa)* the number of both TX and TN genes and only TN genes was found to be higher than *T. cacao, V. vinifera* and *P. trichocarpa* respectively. *B. oleracea* despite having a larger genome (630 Mb) contained less NBS genes than *A. thaliana* (123 Mb) and *B. rapa* (485 Mb) which is may be because of prevalence of higher number of TX, TN and N type genes. TX, TN and TNL genes are prevalent in both conifers and dicots which point out their presence approximately 300 MYA before the divergence of these taxa [54]. The function of TX and TN gene families in plants is unknown yet but their presence in various plant species make them important. Based on the homology of TX and TN genes to the NBS-LRR disease resistance proteins, these shorter proteins act as adapter proteins and have been suggested to play the same role as of Mal and MyD88 (TIR-containing adapter proteins) in the immune system of mammals. In addition, these are also considered as functional proteins and may interact with TNL class for their function in disease resistance [9].

### Influences of tandem duplication and whole genome triplication in *Brassica*species

Plants have experienced more genome duplication events than any other eukaryotes on the earth. After the duplication, genes can follow one of the three functional outcomes, gene loss, neo-functionalization and sub-functionalization [55]. In duplication events, either it is whole genome, segmental or tandem, some genes have more probability to be retained as duplicates. This may be attributed to the certain function of the duplicated genes in particular organism [56, 57]. Approximately 43.3% NBS-encoding genes in *B. oleracea,* 55.7% in *A. thaliana* and 47.1% *in B. rapa* were formed by tandem duplication and distributed in tandem arrays. Tandem duplicated analysis reveal that the rate of tandem duplicated genes was found to be higher in *A. thaliana* than *Brassica* species. After tracing the fate of tandem arrays before or after complete genome triplication, 50 NBS genes were tandem duplicated genes after divergence of *B. oleracea* and *B. rapa* species, representing 31.8% of total NBS-encoding genes in *B. oleracea* genome. There are 83 NBS genes generated after divergence of *B. oleracea* and *B. rapa* species, which represents 40.3% of all NBS genes in *B. rapa* genome. Through analysis of whole genome triplication, 42 *B. oleracea* and 62 *B. rapa* NBS genes were retained on triplicated regions in genomes, representing 26.75% and 30.1% of total NBS genes in *B. oleracea* and *B. rapa* genomes, respectively.

Polyploidy event provide rich genomic resources to study retention and loss of multi-copy genes. Through comparative analysis of NBS-encoding genes among *B. oleracea*, *B. rapa* and *A. thaliana*, we speculated that after WGT of *Brassica* ancestor approximately 16 MYA, orthologous gene pairs for NBS-encoding genes on triplicated regions in genome of *Brassica* ancestor were lost or deleted quickly. The quick loss of paralogs from whole genome duplication might be due to the gene dosage imbalance issue [58, 59]. But after divergence of *B. rapa* and *B. oleracea* approximately 3.7 MYA, NBS-encoding genes in *Brassica* species experienced species-specific gene amplification by mechanism of tandem duplication to adopt environment selection. The increase of gene dosage by tandem paralogs might have some advantage to the plant pathogen defense.

### Disease resistance in *Brassica*species

To environmentally friendly control plant diseases, one of the efficient methods is the employment of genetically disease resistant plants. In the last few years, the studies regarding disease resistance inheritance and molecular cloning of R genes in *Brassica* species made some progress. We found eight conserved genes in *Brassica* species corresponding to the four *A. thaliana* R genes (RPM1, RPS2, RPS4 and RPS5) species in the conserved orthologous region. In present study, we observed one (N type) gene in *B. oleracea* and two (CNL type) genes in *B. oleracea* and *B. rapa* corresponding to RPM1 and RPS2 respectively. Therefore, we propose that these conserved genes in the *Brassica* species may offer gene-to-gene resistance to specific avirulence products from *Pseudomonas syringae* pathogen. *A. thaliana* gene RPS4 (AT5G45250) retained one corresponding orthologous gene in *B. oleracea* (Bol032054) and *B. rapa* (Bra027599) genomes respectively, so we assume that these two genes in *Brassica* species might be race-specific resistant to *Pisi* and *Phaseolicola* subspecies of *Pseudomonas syringae* pathogen. RPS5 retained two copies only in *B. rapa* and these gene copies are species-specific disease resistance genes in *B. rapa* species.

For evolutionary relationship of orthologous gene pairs for race-specific NBS-encoding genes among three species, we compared Ka/Ks values of orthologous gene pairs between *A. thaliana* - *B. rapa* and *A. thaliana* - *B. oleracea* lineages. *A. thaliana* - *B. oleracea* lineage exhibited higher mean Ka/Ks ratios in their orthologous gene pairs than those of *A. thaliana* - *B. rapa* lineage in CNL type NBS-encoding R genes. We conclude that these NBS-encoding genes in *B. rapa* species have undergone stronger negative selection than those in *B. oleracea* species. So, the corresponding NBS-encoding genes in *B. oleracea* species would have experienced stronger evolutionary constraints to adopt changes in the environment. But for TNL type, there are no significant differences between the two species about the orthologous gene pairs. We speculated that these NBS-encoding genes in *B. rapa* and *B .oleracea* species may have undergone different selection pressure to offer resistance to same pathogen or some pathogens may be species-specific pathogens for Brassica species.

## Conclusions

We have identified 157, 206 and 167 NBS-encoding genes in *A. thaliana*, *B. rapa* and *B. oleracea* genomes respectively and total number of NBS-encoding genes in these three species is very close in spite of genome size and WGD/WGT events. Genomic organization and composite phylogenetic analysis facilitate the identification and classification of NBS-encoding genes among *A. thaliana*, *B. rapa* and *B. oleracea*. Expression profiling showing the differential expression pattern of orthologous NBS-encoding genes provides a blueprint for further characterization of these genes in *B. oleracea* and *B. rapa*. The expression profile of different NBS-coding members can be separated into different groups, indicative of functional divergence. Although, orthologous NBS-encoding genes in *B. oleracea* and *B. rapa* are highly divergent but expression pattern divergence among paralogs within a species exceeds the level of divergence among orthologs in each type of NBS-encoding genes. Paralogs might contribute more to functional divergence than orthologs over the evolution of Brassicaceae. Through comparative analysis of tandem duplication and whole genome triplication in NBS-encoding genes among three species, there are fewer paralogous NBS-encoding genes retention after whole genome triplication than those from tandem duplication. So, tandem duplication might play more important influence than whole genome duplication in generation of NBS-encoding genes. We speculated that the quick loss of paralogs from whole genome duplication might be due to the gene dosage imbalance issue. The increase of gene dosage by tandem paralogs might have some advantage to the plant pathogen defense. Our evolutionary studies illustrate that CNL type orthologous genes in *B. rapa* species compared to *A. thaliana* have undergone stronger negative selection than those in *B. oleracea* species compared to *A. thaliana* and opposite to that orthologous genes in *B. oleracea* species experienced stronger evolutionary constraints than those in *B. rapa* species for CNL type R genes. For TNL type NBS-encoding genes, we did not observed significant difference between the two species about the orthologous gene pairs using Mann–Whitney U-test. It is indicated that these orthologous NBS-encoding genes in *B. rapa* and *B .oleracea* species maybe undergone different selection pressure to resist the same pathogen or some pathogens may act as species-specific pathogens for different Brassica species. Through comparative analysis of NBS-encoding genes among *A. thaliana*, *B. rapa* and *B. oleracea*, we hope to explore the evolutionary fate of NBS-encoding genes in *Brassica* lineage after split from *Arabidopsis thaliana* and advance the understanding of disease resistance between *B. oleracea* and *B. rapa* species, which will provide a valuable model for studying functional and evolutionary aspects within the Brassica genus and the crucifer lineage.

## Electronic supplementary material

Additional file 1: Table S1: Information of NBS-encoding genes in *B. oleracea*, *B. rapa* and *A. thaliana.* This table contain the type, distribution of NBS domains, protein full length, subfamily, lists of predicted domains, coding sequences and peptide sequences of NBS-encoding genes in *B. oleracea*, *B. rapa* and *A. thaliana* genomes. (XLS 2 MB)

Additional file 2: Figure S1: Phylogenetic relationship of NBS-encoding genes among *B. oleracea*, *A. thaliana* and *B. rapa*. The Maximum Likelihood tree was constructed by MEGA 5.0 software with 1000 replications. CNL type of NBS-encoding genes was divided into three sub-groups and TNL type was divided into three sub-groups. Each species was shown by different colors. (JPEG 723 KB)

Additional file 3: Figure S2: Heat map representation of NBS-encoding genes in *B. oleracea* and *B. rapa* genomes. I. Heat map representation of NBS-encoding genes in *B. oleracea* genomes. II. Heat map representation of NBS-encoding genes in *B. rapa* genomes. The tissues used for expression profiling are indicated at the top of each column. The genes are on right expression bar. Color scale bar at the bottom of each heat map represents log2 transformed FPKM values, thereby values more than 2, 0 and less than -2 represent positive, zero and negative expression, respectively. (JPEG 1 MB)

Additional file 4: Table S2: List of Tandem arrays of NBS-encoding genes among *B. oleracea*, *B. rapa* and *A. thaliana.* This table contain name, location, gene numbers, gene lists of tandem arrays in *B. oleracea*, *B. rapa* and *A. thaliana* genomes. (XLS 53 KB)

Additional file 5: Table S3: Co-retained tandem duplicated genes of NBS-encoding genes in *A. thaliana* compared to *B. oleracea* and *A. thaliana* compared to *B. rapa* genomes. This table contain co-retained tandem duplicated genes of NBS-encoding genes in *A. thaliana* compared to *B. oleracea* and *A. thaliana* compared to *B. rapa* genomes. (XLS 24 KB)
