# Supplementary figures and images for: Genome-wide comparative analysis of NBS-encoding genes between Brassica species and Arabidopsis thaliana
Source: BMC Genomics. 2014 Jan 3;15(1):3. doi: 10.1186/1471-2164-15-3 (PMC4008172; doi:10.1186/1471-2164-15-3)

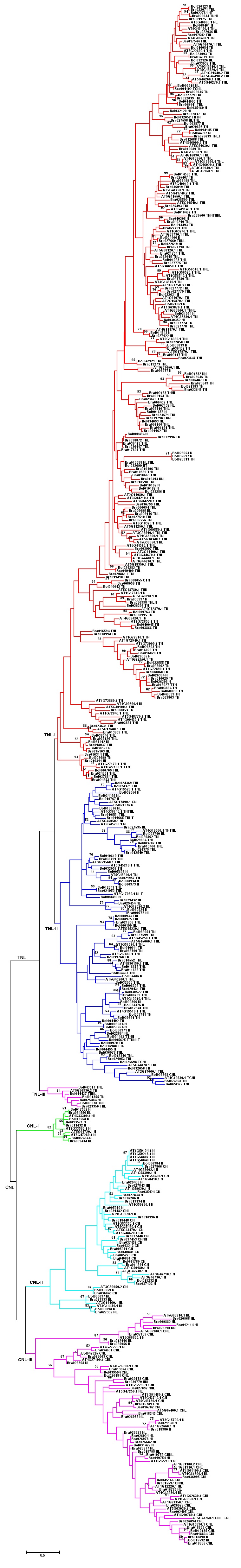

Supplement: Supplementary file 2 — Additional file 2: Figure S1: Phylogenetic relationship of NBS-encoding genes among B. oleracea, A. thaliana and B. rapa. The Maximum Likelihood tree was constructed by MEGA 5.0 software with 1000 replications. CNL type of NBS-encoding genes was divided into three sub-groups and TNL type was divided into three sub-groups. Each species was shown by different colors. (JPEG 723 KB) [file 12864_2013_6997_MOESM2_ESM.jpeg]

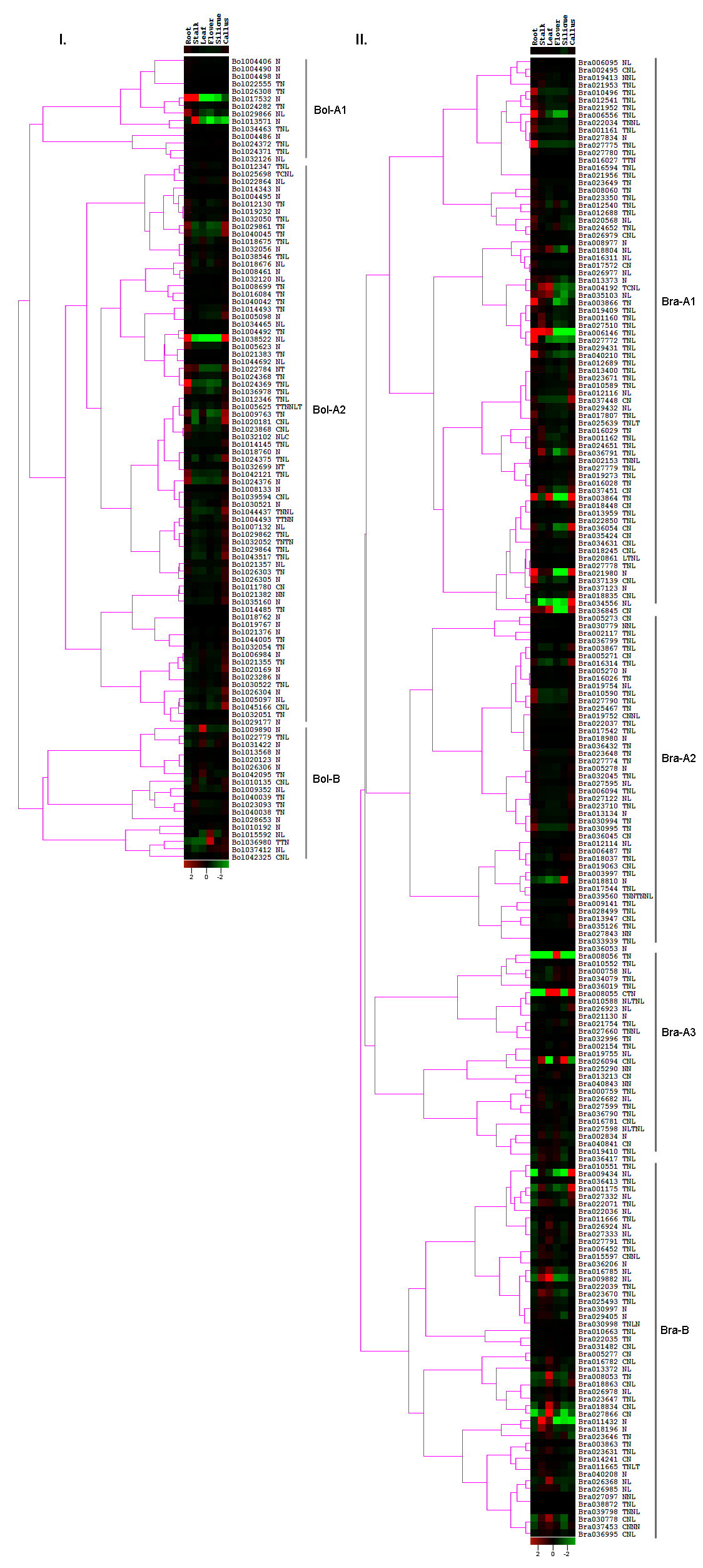

Supplement: Supplementary file 3 — Additional file 3: Figure S2: Heat map representation of NBS-encoding genes in B. oleracea and B. rapa genomes. I. Heat map representation of NBS-encoding genes in B. oleracea genomes. II. Heat map representation of NBS-encoding genes in B. rapa genomes. The tissues used for expression profiling are indicated at the top of each column. The genes are on right expression bar. Color scale bar at the bottom of each heat map represents log2 transformed FPKM values, thereby values more than 2, 0 and less than -2 represent positive, zero and negative expression, respectively. (JPEG 1 MB) [file 12864_2013_6997_MOESM3_ESM.jpeg]
